# PALLiON – PALLiative care Integrated in ONcology: study protocol for a Norwegian national cluster-randomized control trial with a complex intervention of early integration of palliative care

**DOI:** 10.1186/s13063-020-4224-4

**Published:** 2020-04-02

**Authors:** Marianne Jensen Hjermstad, Nina Aass, Sigve Andersen, Cinzia Brunelli, Olav Dajani, Herish Garresori, Hanne Hamre, Ellinor C. Haukland, Mats Holmberg, Frode Jordal, Hilde Krogstad, Tonje Lundeby, Erik Torbjørn Løhre, Svein Mjåland, Arve Nordbø, Ørnulf Paulsen, Erik Schistad Staff, Torunn Wester, Stein Kaasa, Jon Håvard Loge

**Affiliations:** 1 Regional Advisory Unit in Palliative Care, Department of Oncology, Oslo University Hospital, Oslo, Norway; 2 European Palliative Care Research Centre (PRC), Department of Oncology, Oslo University Hospital, and Institute of Clinical Medicine, University of Oslo, Oslo, Norway; 3 Institute of Clinical Medicine, University of Oslo, Oslo, Norway; 4 University Hospital of North Norway, Tromsø, Norway; 5 UiT, The Arctic University of Norway, Tromsø, Norway; 6 Palliative Care, Pain Therapy and Rehabilitation Unit, Fondazione IRCCS Istituto Nazionale dei Tumori, Milan, Italy; 7 Department of Hematology and Oncology, Stavanger University Hospital, Stavanger, Norway; 8 Department of Oncology, Akershus University Hospital, Nordbyhagen, Norway; 9 Department of Oncology and Palliative Care, Nordland Hospital Trust, Bodø, Norway; 10 Department of Oncology and Palliative Care, Førde Hospital Trust, Førde, Norway; 11 Department of Clinical Oncology, Østfold Hospital Trust, Grålum, Norway; 12 Cancer Clinic, St. Olavs hospital, Trondheim university hospital, Trondheim, Norway; 13 Center for Cancer Treatment, Sorlandet Hospital, Kristiansand, Norway; 14 Department of Oncology and Palliative Care, Vestfold Hospital Trust, Tønsberg, Norway; 15 Palliative Care Unit, Telemark Hospital Trust, Skien, Norway; 16 Department of Oncology, Ålesund Hospital Trust, Ålesund, Norway; 17 Institute of Basic Medical Sciences, University of Oslo, Oslo, Norway

**Keywords:** Advanced cancer, Cluster-randomized trial, Palliative care, Integration, Patient-reported outcomes, End-of-life care

## Abstract

**Background:**

Several publications have addressed the need for a systematic integration of oncological care focused on the tumor and palliative care (PC) focused on the patient with cancer. The exponential increase in anticancer treatments and the high number of patients living longer with advanced disease have accentuated this. Internationally, there is now a persuasive argument that introducing PC early during anticancer treatment in patients with advanced disease has beneficial effects on symptoms, psychological distress, and survival.

**Methods:**

This is a national cluster-randomized trial (C-RCT) in 12 Norwegian hospitals. The trial investigates effects of early, systematic integration of oncology and specialized PC in patients with advanced cancer in six intervention hospitals compared with conventional care in six. Hospitals are stratified on the size of local catchment areas before randomization. In the intervention hospitals, a three-part complex intervention will be implemented. The backbone of the intervention is the development and implementation of patient-centered care pathways that contain early, compulsory referral to PC and regular and systematic registrations of symptoms. An educational program must be completed before patient inclusion. A total of 680 patients with advanced cancer and one caregiver per patient are included when patients come for start of last line of chemotherapy, defined according to national treatment guidelines. Data registration, clinical variables, and patient- and caregiver-reported outcomes take place every 2 months for 1 year or until death. The primary outcome is use of chemotherapy in the last 3 months of life by comparing the proportion of patients who receive this in the intervention and control groups. Primary outcome is use of chemotherapy in the last 3 months before death, i.e. number of patients. Secondary outcomes are initiation, discontinuation and number of cycles, last 3 months of life, administration of other medical interventions in the last month of life, symptom burden, quality of life (QoL), satisfaction with information and follow-up, and caregiver health, QoL, and satisfaction with care.

**Discussion:**

Results from this C-RCT will be used to raise the awareness about the positive outcomes of early provision of specialized palliative care using pathways for patients with advanced cancer receiving medical anticancer treatment. The long-term clinical objective is to integrate these patient-centered pathways in Norwegian cancer care. The specific focus on the patient and family and the organization of a predictable care trajectory is consistent with current Norwegian strategies for cancer care.

**Trial registration:**

ClinicalTrials.gov, NCT03088202. Registered on 23 March 2017.

## Background

Major concerns in oncology today are the rapidly increasing complexity of treatment, the steadily augmenting number of treatment lines that are administered, and new agents that increases the demand for anticancer treatment at end-of-life (EoL). Coupled with this is the increasing use of advanced imaging, i.e. computed tomography (CT) and positron emission tomography (PET) scans and magnetic resonance imaging (MRI). About one-third of the cost of cancer care is spent during the patient’s last year of life [1], and costs are expected to escalate rapidly, which threatens the sustainability of the present services [2, 3].

One article examined the rationale for discontinuation of chemotherapy for metastatic non-small-cell lung cancer (NSCLC) and identified a huge variability in these processes, unrelated to time of death [4], as addressed by others [5–7]. This is in spite of patients, families, and oncologists uniformly recognizing the administration of chemotherapy near death as aggressive and as an indicator of poor EoL care [8].

The American Society of Oncology (ASCO) has defined discontinuation of chemotherapy given with a life-prolonging intent in the last 2 weeks of life as a benchmark for improving clinical practice [9]. It is difficult to decide the optimal chemotherapy intensity during the last periods of life at an individual patient level. For example, if the treatment of choice in some patients with chemosensitive solid tumors is a therapy with modest side effects, a high response rate, and good symptom relief, this may be appropriate even if the life expectancy is < 3 months. However, a French registry-based study in > 279,000 patients found no clear pattern between the expected chemosensitivity of different solid cancers and the administration of chemotherapy at EoL [6].

Identifying the appropriate time to stop chemotherapy is challenging, but for the majority of patients chemotherapy should not be given during the last month of life [10, 11]. Generally speaking, it is rare that patients will benefit from chemotherapy in this phase, in fact it may even hasten death [12]. However, the physicians’ attitude and perceptions about oncological treatment, patients’ determination to continue treatment at whatever cost, or the reverse, that the patients’ wishes to spend more time at home and refrain from treatment, are not systematically taken into account in the decision-making process. Another challenging factor is that the patient may not be aware of the prognosis related to the actual diagnosis and stage, either because the prognostic conversation never took place, there was no treatment plan presented that delineated the different scenarios that may occur during the disease trajectory, or because information was provided, but not understood [13–15]. Taken together, these factors may contribute to overuse of anticancer treatment at EoL.

Patients with incurable disease treated with intensive anticancer treatment at EoL more often receive cardiopulmonary resuscitation, mechanical ventilation, or die in an intensive care unit [16]. Further, a study by Prigerson et al. in 2015 showed that use of chemotherapy was not associated with better quality of life (QoL) near death compared with non-use [12]. Interestingly, this was most pronounced in patients with a relatively good level of functioning. This contrasts results showing that a palliative care (PC) approach at an early stage in patients with unfavorable prognosis may improve patients’ QoL and symptom control, may lead to prolonged survival, and give patients and caregivers a more realistic perspective on the disease and prognosis [17–22]. Further, there is documentation that caregivers of patients with advanced cancer are generally more satisfied and report less depressive symptoms with an early introduction of PC [23], although findings are not entirely conclusive across studies.

These and related findings in the wake of the Temel study [24] initiated the current debate and have made international stakeholders advocate a stronger integration of oncology and PC for patients with incurable cancer [8, 25–27]. Despite a rapid development of PC services during the last 15–20 years, there is still a distinct separation between oncology and PC in Norway as in other Western countries. This infers that the potential for optimal care during the last phase of life for patients with advanced cancer has not been fully explored, despite the documented advantages.

Information given to cancer patients and caregivers about the disease and its treatment is important at all stages of disease and is crucial in relation to PC in order to facilitate shared decision-making, and improve satisfaction with care, patients’ sense of control, QoL, and communication between healthcare providers and patients and caregivers. As PC also aims to improve the QoL of caregivers [28], waiting to include caregivers until patients are in their last weeks or days of life may not adequately address patient or caregiver distress [13, 29]. However, results from studies on integration of oncology and PC from other countries such as the UK and United States, may not be directly transferable to Norway, given the different organization of healthcare, reimbursement issues, and indications for hospice use. Further, no national trials have been conducted yet.

On this background, we launched the PALLION study, a cluster-randomized controlled trial (C-RCT) in 12 Norwegian hospitals. The long-term objective is to improve the quality and efficacy of cancer care for patients with advanced cancer who receive chemotherapy. This will be achieved by means of a complex intervention consisting of the development and implementation of specific patient-centered care pathways (PCCPs) that delineate the treatment and care trajectory, make early referral to PC compulsory alongside anticancer treatment, and contain systematic registration of patient- and caregiver-reported outcomes (PROs) during treatment and follow-up. A prerequisite to succeed with the integration of oncology and PC is the specifically developed educational program for physicians to be completed before patient inclusion.

The novelty of this study is that it represents a change of today’s oncological practice by implementing time-based referrals to PC as opposed to and in addition to today’s ad-hoc and needs-based referral practice. The primary study objective is to investigate the effects of this complex intervention by comparing the use of chemotherapy in the last 3 months of life between the intervention and control groups. Secondary objectives focus on the use of medical interventions at the EoL, patient- and caregiver-reported outcomes, and direct costs.

### Patient-reported outcomes (PROs)

In 2006, the U.S. Food and Drug Administration (FDA) proposed the term patient-reported outcome measures (PROMs) for all measures that can best, or only, be assessed by asking the patients [30]. PROs denote an umbrella term covering the patient’s perspective on physical and psychological wellbeing, symptoms, and treatment effects [31], hence supplement clinical observations. The recognition of PROs as independent outcomes is consolidated by the Consolidated Standards of Reporting Trials (CONSORT) PRO extension statement [32] developed to improve the reporting of PROs.

Performance status and tumor burden are important predictive factors for effect of chemotherapy and survival, and closely linked to symptom burden and patient wishes. Thus, a patient-centered focus with systematic evaluation of PROs and preferences should be part of any treatment, to optimize care, particularly so as tumor-directed treatment becomes less efficient or is discontinued. Nevertheless, it is well documented that systematic symptom assessment is not routine in oncological care [33–35], although frequently used in clinical trials due to the FDA requirements [36]. Unsystematic use of PROs may in part explain inadequate symptom management. This applies to pain as well as other physical and psychological symptoms that are highly prevalent among patients with cancer, such as appetite loss, constipation, dyspnea, fatigue, anxiety, depression, and overall QoL.

Traditionally, PROs have been collected by paper forms that are perceived as cumbersome to administer, use, and store, all commonly cited barriers explaining the infrequent use of PROMs in clinical practice [33, 34]. Electronic measures, e-PROMs, can use different software and platforms, permit immediate use of real-time data in clinical consultations, and direct follow-up of patients outside hospital. E-PROMs can be programmed with a flexible and dynamic content that automatically selects the most appropriate questions to a given patient, based on a few initial screening items. They may be integrated with medical data from sources such as laboratory, imaging, and electronic health records. Studies on the use of e-PROMs in oncological care have shown that this facilitates communication [18, 37, 38], results in better patient wellbeing [27, 39], is well achieved by patients and clinicians [35], and might result in more efficient and focused consultations [40, 41]. One C-RCT demonstrated better physician satisfaction as they became aware of patient symptoms that had not been addressed [42]. A secondary analysis from a RCT with one group registering symptoms regularly suggested a potential survival benefit, but needs further investigation in larger samples [43]. Systematic electronic collection of PROs is part of PALLiON.

### Care of the caregiver

Informal caregivers frequently serve as the patient’s main source of support. In the United States, approximately 10% of about 40 million caregivers report caring for a person with cancer [44, 45]. As cancer care has shifted towards more outpatient and home-based care, the strain on caregivers is likely to increase.

Caregiving can be rewarding and positive, but the burden of caregiving might be substantial and many may not be prepared for or able to do this. Further, the caregiving experience related to advanced cancer differs from caregiving in other chronic conditions by the fluctuating, unpredictable, and often rapidly deteriorating condition of the patient. Reduced QoL and distressing symptoms such as insomnia, fatigue, anxiety, depression as well as financial difficulties are commonly experienced among caregivers [46]. Importantly, the individual caregiver vulnerability is highly associated with the perceived extent of caregiver burden [47].

One article states that early PC is an example of how to improve or maintain quality of healthcare delivery for patients and caregivers at a lower cost [48], but studies evaluating caregiver QoL, satisfaction with care, and emotional distress are equivocal. One Norwegian study [49] showed an overall positive effect on QoL, in line with studies of caregivers of patients with a poor prognosis treated by specialty-trained PC clinicians [50–52].

Two consecutive meta-analyses, covering 79 RCTs, examined the effect of different interventions on outcomes in caregivers of patients with cancer [46, 53]. The first reported small-to-medium effects on caregiver burden, coping, QoL, and self-efficacy [53], while the second concluded that caregiver interventions should be flexible and adapted to the actual situations [46]. Results from qualitative analyses indicate that provision of early PC might improve caregiver satisfaction and reduce caregiver distress [23, 54, 55]. As these interventions varied largely, no firm conclusions about which is the best approach can be drawn.

Involvement and support of caregivers are regarded key components of modern cancer care, as explicitly stated in the updated ASCO Clinical Practice Guideline on integration of PC and oncology [56]. Nevertheless, caregiver perspectives and experiences are often not adequately addressed in the consultations [47]. Hence, caregiver follow-up is systematically implemented in the PCCPs in PALLiON.

### Patient-centered care pathways (PCCPs)

Many patients perceive the healthcare system as fragmented; complaints about uncoordinated care are common [57], particularly so in transitions from one healthcare level to another [58].

The European Pathway Association (EPA) defines standardized care pathways (SCPs) as “a complex intervention of the mutual decision-making and organization of care processes for a well-defined group of patients during a well-defined period” [59]. Thus, SCPs may be a method of organizing complex care processes. In integrated oncological and patient-centered care, formalized as an SCP, healthcare professionals (HCPs) from different professions and at different locations work together to achieve common goals, i.e. better patient treatment and care. An SCP can structure individualized patient-centered care with predictable trajectories, with evidence-based diagnostics, treatment, symptom management, plus shared decision-making and family involvement. The pathway can be tailored to the individual patients in a PCCP, as in PALLiON.

The structure and content of PCCPs decide the quality of care. PCCPs may be a work plan for HCPs to optimize the use of competence and resources, i.e. providing the right competence to the right patient at the right time in the right place. Further, PCCPs may facilitate seamless transitions within and between hospitals by formalized collaboration across specialties and clarifying responsibilities. Electronic PCCPs give immediate access to useful resources such as treatment guidelines, toxicity criteria, what to address in prognostic conversations, how to promote shared decision-making, etc., all of which ensures a more uniform and quality-based interaction with the patient. A recent Lancet Commission paper proposes PCCPs as the method of choice for the integration of anticancer and patient-centered care [8], inspired by the use of PCCPs in PALLiON.

Despite the relatively consistent results from RCTs and observational studies of beneficial outcomes of early integration of PC and oncological care, it is noteworthy that designs and models of integration vary largely. Further, few studies have tested a full integration according to the criteria set forth by Leutz [60]. This indicates that there is no one right model of integration that can be implemented anywhere, and that organizational, cultural, and health policy aspects must be considered to succeed.

On this background, we have launched the national cluster-randomized, non-blinded multicenter parallel group PALLiON study, with the overall, long-term objective to integrate oncological care and PC in Norwegian cancer clinics, to improve the quality and efficacy of cancer care. PALLiON is a complex intervention containing education of physicians, implementation of PCCPs with predictable care trajectories of anticancer treatment, and patient- and caregiver-centered care with early time-based referrals to PC and systematic symptom registrations. Primary and secondary study outcomes are use of chemotherapy during the last 3 months of life and patient- and caregiver-reported outcomes.

## Methods

The trial is developed according to the Standard Protocol Items: Recommendations for Interventional trials (SPIRIT) checklist (Additional file 1).

### Design

The present study is designed as a parallel group, national, multicenter, C-RCT.

### Study objectives and endpoints

The primary study objective is to investigate the effects of the complex intervention by comparing the use of chemotherapy in the last 3 months of life between the intervention and control groups. This will be assessed using the following primary endpoint: the proportion of patients who receive chemotherapy during their last 3 months of life; and, by the first secondary outcomes, the number of cycles administered in this period, time from initiation of last cycle to death, and average time from discontinuation of last cycle to death.

Additional secondary objectives focus on: (1) the use of medical interventions / treatments at the EoL; and (2) patient- and caregiver-reported outcomes. The first will be assessed by the proportion of patients who receive this, as with the primary outcome. Patient- and caregiver-reported outcomes will be assessed every other month using well-validated questionnaires. These endpoints are defined as average area under the curve of follow-up assessments for each outcome.

Exploratory analyses will also investigate potential differences in length of survival between the two arms and examine potential differences in direct costs of care (Table 1).

**Table 1:** Primary, secondary, and explorative objectives, and related endpoints and assessments

	Objectives	Endpoint	Assessment method
Primary	To compare use of chemotherapy in the last 3 months of life in the control and intervention groups	Proportion of patients who receive chemotherapy at EoL	Numerical and descriptive data from HCP registrations/e-CRF, last 3 months before death
Secondary	To examine the administration of chemotherapy last 3 months, medical interventions/treatments at EoL, after discontinuation of chemotherapy	Number of chemotherapy cycles, initiation, and discontinuation Proportion of patients who receive other medical interventions, i.e. concomitant medication and artificial nutrition	Numerical and descriptive data from HCP registrations/e-CRF, last 3 months of life, and after discontinuation of chemotherapy
	To compare PROs	Patient-reported symptom burden, QoL, anxiety/depression, satisfaction with information	EAPC basic dataset. EORTC-QLQ PAL15, PHQ-9, GAD-7, EORTC QLQ-INFO25
	To compare caregiver-reported outcomes	Caregiver-reported health, QoL, and satisfaction with care	SF-36, FAMCARE
Explorative	To examine length of survival	Length of survival from start of last line of chemotherapy	HCP registrations, Cause of Death Registry
	To examine direct costs	Costs of healthcare, oncology/palliative units	Length of stay, number of hospitalizations, anticancer and other medical interventions

*e-CRF* electronic case report form, *EoL* end-of-life, *HCP* healthcare provider, *PRO* patient-reported outcome, *QoL* quality of life

### Sample size

Sample size calculation was based on the primary endpoint: the proportion of patients who receive chemotherapy during their last 3 months of life (Table 1). This took into consideration the predetermined number of 12 as the total number of clusters, i.e. hospitals, that was chosen for contingency/feasibility reasons and allowing for intra-cluster correlation coefficient (ICC) in the range of 0.06–0.09, which is usually considered appropriate for process outcomes. In addition, calculations were performed to provide 80% power at the two-sided 5% level to detect a 25% difference in chemotherapy use between the two arms, i.e. 20% versus 45% in the treatment and control groups, respectively [61]. The choice of the clinically relevant treatment effect used for sample size calculation (25% difference for chemotherapy administration) was based on both clinical judgment and previous data [62, 63]. Effects on power increase due to adjustment by baseline measurements and other covariates (see statistical analysis section for the full list of covariates proposed) were also considered, allowing for correlation coefficient between outcome and adjustment covariates (r_adj_) up to 0.4. Table 2 reports sample size per treatment arm for various values of ICC and (r_adj_).

**Table 2:** Sample size per treatment arm for various values of ICC and (r_adj_)

			**r**_**adj**_		
	*0.4*	*0.3*	*0.2*	*0.1*	*NO*^a^
**ICC**					
*0.06*	102	120	138	150	150
*0.07*	126	150	180	198	204
*0.08*	156	204	258	**300**^b^	312
*0.09*	210	318	468	630	714

*ICC* intra-cluster correlation coefficient, *r*_*adj*_ correlation coefficient between outcome and adjustment covariates

^a^No adjustment for baseline covariates

^b^Required no. of patients per arm

A sample size of 300 patients in each arm, with a total of 600 patients, was chosen as it accounts for an ICC up to 0.08 even with a low r_adj_ (no less than 0.1) and for an ICC up to almost 0.09 in case of r_adj_ no less than 0.3. Due to an expected attrition rate up to 12%, 340 patients per arm (680 in total) will be enrolled.

The 12 hospitals were grouped into three strata according to size of catchment area, to decrease the chance of imbalance in cluster characteristics between trial arms. Each cluster consisted of four hospitals: small (69,000–136,000 inhabitants); medium (169,000–282,000 inhabitants); and large (300,000–492,000 inhabitants) (Table 3). This procedure is considered a reliable and easy to measure proxy for other cluster characteristics, potentially impacting on the study outcome. Effect of the variability in cluster size (mean 50, SD 19.5, CV [Coefficient of variation] = 0.39) was calculated through the approach proposed by Van Breuckelen [64]. For CV = 0.39, this method indicates low inflation factors which are in the range of 2.8%–2.2%, for ICC in the range of 0.06–0.09, respectively [64]. The role of the correlation coefficient in sample size formula for binary outcomes and when adjusting for baseline factors is explained by Hemming et al [65]. Sample size calculations in Table 2 were undertaken using the module clustersampsi [65] implemented in the statistical package Stata (Stata Statistical Software, Release 14, Stata Corp., College Station, TX, USA).

**Table 3:** Presentation of stratification, randomization results, and sample size at cluster level

Intervention arm				Control arm			
Hospital	Stratification	Inhabitants	Patients	Name	Stratification	Inhabitants	Patients
Stavanger University Hospital, Stavanger	1	352,650	85	Akershus University Hospital, Nordbyhagen	1	490,000	85
Oslo University Hospital	1	340,000	85	St. Olavs Hospital/Trondheim University Hospital, Trondheim	1	300,000	85
Østfold Hospital Trust, Grålum	2	282,600	50	Sørlandet Hospital, Kristiansand	2	170,000	50
Vestfold Hospital Trust, Tønsberg	2	233,000	50	Telemark Hospital Trust, Skien	2	169,000	50
Nordland Hospital Trust, Bodø	3	136,000	35	Førde Hospital Trust, Førde	3	109,000	35
Ålesund Hospital Trust, Ålesund	3	104,000	35	University Hospital of North Norway, Tromsø	3	125,000	35
**Total patients**			**340**	**Total patients**			**340**

### Eligibility criteria

The randomization process in C–RCTs consists of two levels: first, the clusters where the randomization takes place, *here: the hospitals*; then, the individual participants, *here: cancer patients*.

### Inclusion criteria: cluster level

Three explicit eligibility criteria apply to hospitals on the cluster level:

1. The hospital should have established both oncology and PC programs:
  a According to Norwegian healthcare policy, the latter implies that the program consists of an ambulatory specialist PC team consisting of at least one physician and one nurse. The team serves the hospitals’ inpatients and may also engage in extra-mural activities;
2. The hospital should have PC programs including EoL care;
3. The hospital should have a local catchment area (as opposed to being a referral center only), meaning that a defined proportion of their cancer patients receive all cancer treatment in this hospital and are not transferred shortly after diagnosis for further treatment and follow-up elsewhere.

Further, it was an explicitly stated criterion for funding in the call that all four Norwegian health regions should be represented. Thus, this is considered on the cluster level of the trial.

### Study population

#### Inclusion criteria: participant level

##### Patients

At the second level of the C-RCT, the following eligibility criteria apply to patient inclusion:

- A verified metastatic or locally advanced cancer of the upper gastrointestinal (GI) tract, lower GI tract, pancreas, liver, breast, bladder, prostate, kidney, cholangiocarcinoma, or malignant melanoma;
- Defined as a patient in need of PC with an anticipated life expectancy of < 12 months;
- Scheduled to start the anticipated last line of chemotherapy (definition: tumor directed systemic therapy). Time of inclusion that is based on the decisions about last line of chemotherapy, varies by diagnosis, and is equivalent to first line in some diagnoses;
- Age > 18 years;
- Fluency in written and oral Norwegian;
- Physically and cognitively able to provide written consent, based on clinical criteria, e.g. not too frail or having signs of disorientation, confusion, attention deficit, poor coordination;
- Scheduled to receive all oncological and specialized palliative treatment at the participating hospital, i.e. residing in the local area of the including hospital;
- WHO performance status 0–2.

Because gynecological and lung cancer patients receive anticancer treatment at the departments of lung and gynecology in most hospitals, these diagnoses were not included.

##### Caregivers

In this study, a caregiver is defined as “the person in a close supportive role sharing the illness experience with the patient, according to the patient.” Thus, this may not necessarily be a relative, although it most often is. Caregivers will be identified by asking the patient whom he or she identifies as the primary caregiver. Study personnel will then ask permission from patients who consent to the study if it is OK to ask his/her caregiver to participate as well. If a caregiver declines to participate, the patient can still be included and followed in the study.

The following set of eligibility criteria applies to the inclusion of caregivers:

- Defined by the patient as the primary caregiver;
- Age > 18 years;
- Fluency in written and oral Norwegian;
- Physically and cognitively able to provide written consent, based on clinical criteria, e.g. not too frail or having signs of disorientation, confusion, attention deficit, poor coordination.

#### Exclusion criteria: participant level

- Any serious psychiatric diagnosis (e.g. psychotic, bipolar disorder), substance abuse, or cognitive impairment as judged by standard clinical criteria (disturbed consciousness, disorientation to time/place and attention deficits) that precludes completion of PROs, at the discretion of the attending physician/study personnel;
- A cancer diagnosis other than the ones specified in the inclusion criteria (patients);
- Multiple malignancies (patients);
- Already included and followed in a PC program (all);
- Serious substance abuse (all).

#### Randomization, blinding, and study monitoring

Randomization was performed within each stratification group using a web-based randomization system developed and administered by the Unit for Applied Clinical Research, Institute of Cancer Research and Molecular Medicine, Norwegian University of Science and Technology (NTNU), Trondheim, Norway. The procedure was concealed for all study personnel. The allocation of hospitals to the intervention and control arms is shown in Table 3.

### The complex intervention

The complex intervention in the PALLiON study consists of three separate, but highly integrated, parts that will be performed in the six hospitals randomized to the intervention arm:

- An educational program for involved oncologists and PC physicians and nurses;
- A patient care pathway, mapping treatment and care activities from inclusion through follow-up, thereby consolidating an integration of PC and oncology;
- Systematic symptom assessment in all consultations, preferably computer-based.

### The structured educational program

The aims of the educational program are to provide the participants—i.e. oncologists, doctors under specialization, PC physicians and residents—with sufficient competence, theoretical knowledge, understanding, and practical skills to make a complete integration of oncological care and PC in the PCCPs. The content of the program is specifically selected as they represent transitions in a disease and treatment trajectory, e.g. in relation to discontinuation of treatment and disease progression, and that are often perceived as difficult by clinicians, such as communication of prognosis and breaking bad news. Thus, the content of the lectures correspond with the organization of care and the specific content at each step of the PCCSs. Hence, the program is to be completed before patient inclusion in the PALLiON study, with a mandatory confirmation that it has been completed.

The educational program takes place at each of the six intervention hospitals. It consists of classroom lectures, Internet teaching videos, group work, and 1 day with communication skills training focusing on specific communication tasks related to integration of oncology and PC. The first of the eight lectures is a thorough introduction lecture presenting the scientific basis, organization, methods, and objectives of the PALLiON study. The following seven focus on central issues related to palliative treatment. They have an overall duration of 3 h, use the PowerPoint format, and are taped with voice-over to make them available in the e-learning program (see below). All lectures are given by experienced physicians who are specialists in oncology and/or PC and with long-standing clinical experience. The content is based on evidence-based treatment guidelines on chemotherapy and radiotherapy endorsed by the ASCO [66] and the American Society for Radiation Oncology [67], symptom assessment, and relevant literature related to each subject. The following areas are covered: (1) PALLiON—care pathways and integration of PC and oncology; (2) the educational program—objectives, structure, content, short info about Eir; (3) using Eir in clinical practice, symptom assessment; (4) prognostication, prognostic tools; (5) palliative radiotherapy; (6) palliative chemotherapy; and (7) EoL care.

The e-learning program follows the structure of the one at the Faculty of Medicine at the University of Oslo and has an overall duration of 3 h. Four videos are used, in which a young patient with breast cancer is followed. An experienced nurse plays the patient while an experienced oncologist and an experienced PC physician feature the consulting physicians. The first video starts when the patient comes to the oncologist after having completed third-line chemotherapy a while ago. She is then followed towards death, with the films covering: prognostic information; treatment decisions, e.g. cessation of therapy; introduction of PC; and talking about death and dying. The videos are supplemented with exercises developed to reflect upon the contents of the films, learning objectives, presentations of specific communication skills, and references to relevant literature, intended for group or individual work at each intervention site.

The practical communication training is divided into two areas: (1) skills training; and (2) hands-on guidance. The first consists of local courses for small groups at the participating sites, focusing on specific communication challenges such as starting a discussion of prognosis and treatment intention, elucidating the patient’s preferences and provision of relevant information to patients facing termination of anticancer treatment and EoL. These are led by two experienced psychologists from the PALLiON education team and lasts for 1 full day. The hands-on guidance takes place after skills training. A local coach attends one consultation as an observing participant and gives the oncologist or PC physician feedback afterwards.

The structured educational program will be completed at all sites before patient inclusion and starts in the fall 2016. All lectures and instruction videos are available at the restricted access part of the PALLiON website and all participants in the intervention arm are encouraged to use this material for continuous discussions and updates. Access for participants in the control arm will be provided after study closure.

### Eir for systematic electronic symptom assessment

Eir is an Internet-based communication platform introducing a standardized way of assessing and immediately using the patients’ self-report of symptoms in clinical consultations. The intention behind the development of Eir was to improve symptom management by:

- Systematic symptom assessment and management;
- Timely presentation of symptoms to HCPs, regardless of location of patients and HCPs (oncology unit, outpatient unit, home, hospice, etc.);
- Facilitating patient-centered communication by immediate presentation of the most bothersome symptoms and problems;
- Options for providing evidence-based decision support based on patients’ symptom scores and the doctor’s clinical evaluation.

Eir is a HTML-based symptom assessment tool incorporating a limited amount of decision support based on evidence-based treatment guidelines. It is primarily based on PROs assessing subjective symptoms, functioning, and QoL. The system consists of multiple modules with the patient and doctor modules being used in the PALLiON study.

The patient module consists of multiple levels, starting with a general question about wellbeing, thereby mimicking a common introduction to a consultation. This question is followed by 19 common cancer symptoms to be ticked by the patient if present. These are identical to the ones in the European Association for Palliative Care (EAPC) basic dataset [68] supplemented by a few items of particular relevance for chemotherapy side effects, e.g. numbness in in fingers/toes. Subsequent questions are then presented to further evaluate and characterize the ticked symptoms for each patient. Most of the ticked symptoms are first scored on a numerical rating scale (NRS) of 0–10 and followed by in-depth questions depending on the NRS scores. The follow-up questions are taken from well-validated and frequently used questionnaires, such as the Edmonton Symptom Assessment System (ESAS) [69], Brief Pain Inventory (BPI) [70], Subjective Global Assessment for nutritional status (SGA) [71], Patient Health Questionnaire-9 (PHQ-9) [72], and European Organisation of Research and Treatment of Cancer Quality Of Life Questionnaire Core 15 Palliative (QLQ-C15-PAL) [73].

Patients respond to the questions on a tablet that is connected to WiFi. The responses are immediately transferred and stored at a secure server that can be accessed by the HCP who logs on to the doctor module on a free-standing computer (not connected to the hospital’s intranet). The most recent PROs are presented, supplemented by a graphical display showing the development of symptoms over time if the patient has been seen before. Furthermore, treatment advice can be viewed on the physician’s computer screen. For example, suggestions to start pain medication, change the dose, switch to another drug, adding laxatives, etc. may pop up, based on the patient’s present and previous pain scores. Current performance status and weight changes are displayed on top of the screen.

The Eir system was evaluated and approved by HEMIT [74] in 2015. The thorough evaluation focused on security and risks, in the form of a risk and vulnerability analysis. No security violations or serious risks were detected. The use of Eir will be performed in accordance with regulations and standards at each of the six intervention hospitals that will use Eir as part of the care pathways.

### The patient-centered care pathways

**Fig. 1:**
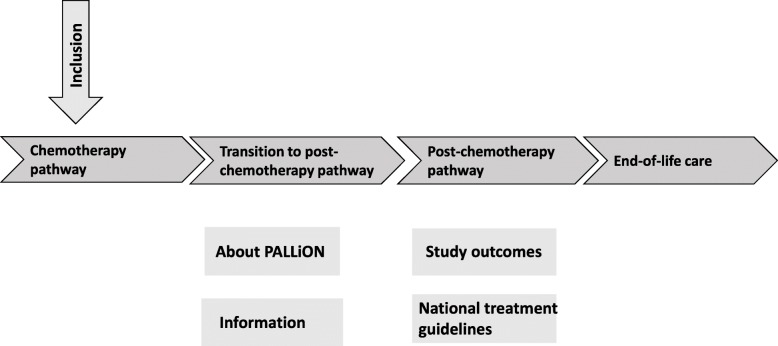
The patient-centered care pathways and supplemental information *. * arrows and boxes are interactive and contain essential aspects and additional information for each step of the pathway

A PCCP in PALLiON means that the planned treatment trajectory for *the individual* patient is outlined and presented to the patient and caregivers shortly after inclusion in the study. The PALLiON pathways are easily accessible on all hospital and free-standing computers by a shortcut key, to the front page (Fig. 1). The pathways are developed for everyday clinical use in that they guide the physicians’ and other healthcare providers’ actions and responsibilities in the four sub-pathways depicted by the white arrows in Fig. 1. The content corresponds with national recommendations for treatment and follow-up for the respective diagnostic groups [75], and the former and updated national strategies for cancer in Norway [76, 77].

**Fig. 2:**
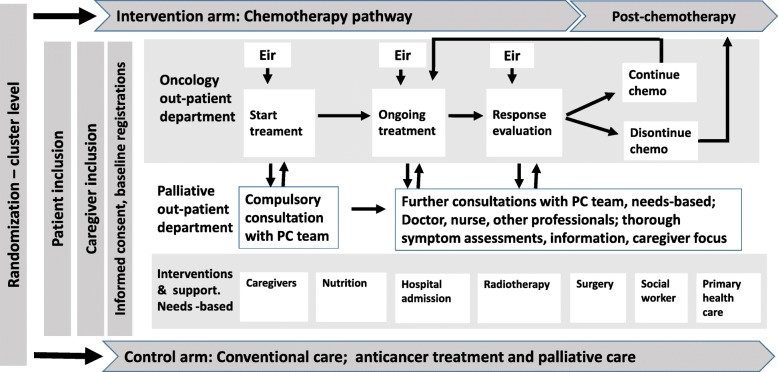
The chemotherapy pathway

For each of the four sub-pathways or phases in Fig. 1, i.e. chemotherapy, transition to post-chemo, post-chemo, and EoL care, the tasks of the involved physicians are clearly defined, as illustrated in the chemotherapy pathway in Fig. 2. The pathways will ensure more standardized treatment decisions, systematic evaluation of treatment effects and side effects according to established criteria, i.e. CTCAE Common Terminology Criteria for Adverse Events [78] and RECIST Response Evaluation Criteria In Solid Tumors [79], regular symptom monitoring using Eir, and a structured integration between the oncologist and the PC team at defined timepoints. They include different clinical scenarios that may become relevant during the last year of life, e.g. rapid disease progression, frailty, treatment toxicity, etc. Involvement of patient and caregiver is specifically focused in all consultations. The high level of details aims to improve the predictability of the disease trajectory during the last year of life and allows for adaptations based on continuous clinical considerations and patients’ and caregivers’ preferences as death comes closer.

The chemotherapy pathway as shown in Fig. 2 consists of carefully selected factors that together constitute a predictable trajectory that integrate oncological and PC for patients and caregivers.

These key factors encompass:

- Clinical outcomes and PROs as these variables constitute an important premise for the decision-making regarding start, pause, or discontinuation of chemotherapy and/or other treatment strategies;
- Formalized, continuous collaboration between oncology and PC starting at study inclusion, with a compulsory PC consultation for all patients and caregivers;
- Defined responsibilities, by explicitly stating who is the primary responsible physician, the oncologist or the PC physician, depending on where the patient is in the trajectory;
- Systematic inclusion of patient preferences and active participation in decision-making;
- Involvement of family members upon start of last line of chemotherapy, and onwards;
- Explicit, shared, and delegated patient and caregiver responsibilities and information;
- Systematic symptom assessment with Eir as part of the standardized care pathway;
- Systematic transfer of information between the hospital, general practitioners, and the community health services;
- Multi-professional collaboration with referrals as needed.

All patients in the intervention arm will have regular follow-up during the disease trajectory by the PC team both within and outside of hospital, with regular assessments of PROs (symptoms, psychological distress, social support) with phone calls approximately every other week, PC consultations as needed, and assessment of need for home care or nursing home admissions.

### The control arm

Patients in the control arm will be included when coming for start of what is the anticipated last line of chemotherapy as the same inclusion criteria apply as for patients in the intervention arm. Anticancer and other medical treatment, PC, and patient follow-up will be conducted according to established guidelines. Symptom management, contact with the hospitals’ PC teams, referral to other HCPs, care and contact with caregivers, and use of healthcare services will follow common practice. HCPs will register these activities in the e-CRFs (Table 4).

**Table 4:**
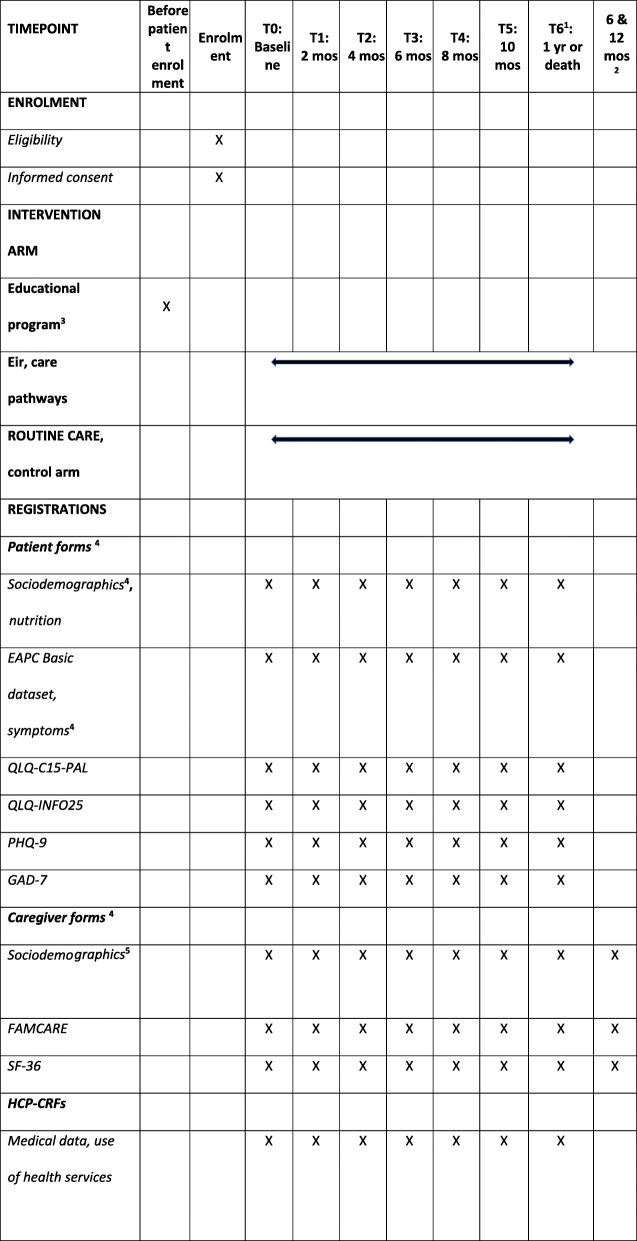
Study schedule, according to the Standard Protocol Items: Recommendations for Interventional Trials (SPIRIT) guidelines

^1^Self-reported data are collected up to seven times during the first year, or until the patient’s death or study withdrawal, whichever comes first. As final date for inclusion is 31 December 2020, patient follow-up will continue in 2021

^2^Caregivers receive two forms at 6 and 12 months after the patient is dead, provided that they consent to continue in the study. Thus, caregiver follow-up may continue in 2022

^3^Educational program for physicians to be completed before patient enrolment

^4^All study forms are similar in both arms

^5^Sociodemographic data that are unlikely to change over time are only registered at baseline

### Withdrawal criteria

Patients may withdraw from the study at any time at their own request. They receive information that they do not need to explain the reason for withdrawal. If a patient decides to discontinue the study, we confirm that the request is received. We will also ask if the patient consents to the use of data that have already been collected. If not, the previously collected data are deleted.

### Trial procedures

Table 4 shows the study timeline and all data registrations from enrolment to end of study, according to SPIRIT guidelines.

### Data collection and statistics

#### Data registration by study personnel

##### Patients

An e-CRF, developed by the Unit for Applied Clinical Research at NTNU, is used for registration of clinical data, every other month until death or for 1 year. This is the responsibility of the study nurses and Principal Investigators at the participating sites. The first questions of the initial CRF contain confirmation of written informed consent and whether the caregiver can be asked to participate. These items are not replicated in subsequent registrations. The clinical variables are: height; weight; cognition (date, time, day, backwards spelling); co-morbidities; principal cancer diagnosis and date of diagnosis; stage; presence; site and number of metastases; previous and present anticancer treatment (within the past 2 weeks); Karnofsky Performance Status (KPS) score [80]; regular use of medication; current use and dosage of opioids; and the following biomarkers if within the past 7 days: C-reactive protein; hemoglobin; leukocytes; thrombocytes; albumin; GT (Gamma-glutamyltransferase); LD (Lactate-dehydrogenase); creatinine; and contact with the community healthcare services and general practitioner. The same set of variables are registered during follow-up, supplemented with detailed registrations of all anticancer treatment, other medical interventions, change of medication, imaging (X-ray, MRI, CT and PET scans), transfusions, and more administrative data, such as department category for treatment, i.e. palliative or general oncology unit, in- or outpatient status, and emergency or other hospital admissions.

##### Caregivers

There is no medical CRF for caregivers, but a brief CRF on background data, co-morbidities, and use of healthcare services.

##### Statistics

The design makes it possible to evaluate the effect of an early integration of oncology and PC at the institutional and patient levels across intervention and control arms, by comparing the use of chemotherapy in the last 3 months of life.

Analyses on the primary outcome will be performed when there are 300 patients in each arm with data on chemotherapy use in the last 3 months of life, which is the basis for the power estimation. Standard descriptive statistics will be used for background variables. Further, data will be analyzed by the intention-to-treat approach; mixed effect regression models will be used to analyze both primary and secondary endpoints in order to account for clustering. Logistic and linear regression models will be used respectively for binary and continuous outcomes and all models will be adjusted for potentially relevant baseline covariates at patient level, i.e. sex, age, diagnosis, performance status score, and baseline symptom scores. The analysis of the main outcome will also be adjusted by size of the catchment areas, which is the randomization stratification factor.

Survival analysis methods, i.e. Kaplan–Meier curves and Cox proportional hazards models with shared frailty terms to account for clustering and Kaplan–Meier curves, will be employed in order to model time to death. Sensitivity analyses to different methods will be applied to handle missing data in the secondary PROs: complete case analysis and multiple imputations. This also applies to patient- and caregiver-reported outcomes. Paper forms will be used to collect medical data and PROs for comparisons across arms.

Further, a potential effect of the intervention at each of the centers in the intervention arm may be evaluated at a later stage as an explorative outcome by comparing post-intervention data with historical data, e.g. on use of chemotherapy and number of readmissions/emergency admissions in the very last stages of life as appropriate.

The CONSORT recommendations for reporting RCTs will be followed. The CONSORT recommendations for reporting C-RCTs will be followed. IBM SPSS Statistics for Windows, version 25.0 (IBM Corp., Armonk, NY, USA) and STATA (Stata Corp. LCC, College Station, TX, USA) will be used.

### Data collection, patient registrations

A consensus-based set of key variables to describe or classify a PC cancer population has been requested for years and resulted in the EAPC Basic Dataset published in 2019 [68]. The second to final version of this dataset is used in this study, without grading of dementia, chronic obstructive pulmonary disease, and heart failure.

### Patient-reported outcome measures

Patient- and caregiver-reported data are collected on paper forms, sent by mail from the Trial Office every other month (Table 4). Returned forms will be stored there according to confidentiality regulations.

### The EAPC basic dataset: background information and symptoms

This part of the patient form is similar to the EAPC basic dataset [68] with a few adaptations to the present study. The form contains 14 questions: sociodemographic variables, i.e. date of birth, date of consent, and marital, living, educational, and working status, followed by ethnicity, use of tobacco and alcohol, and five questions on height and weight, nutritional status, and intake modified from the SGA [71]. For subsequent forms, one question about use of healthcare services since inclusion has been added, whereas general background items that are unlikely to change between assessments have been dropped, to reduce respondent burden.

This form contains 12 frequent cancer-related symptoms: pain; drowsiness; tiredness; nausea; appetite; dyspnea; depression; anxiety; wellbeing; sleep; constipation; and vomiting. The period is right now, and patients are asked to rate the intensity on a NRS of 0–10 (0 = no pain/no dyspnea, etc. to 10 = worst imaginable pain/dyspnea, etc.).

### EORTC QLQ-C15-PAL

The QLQ-C15-PAL [73] is a shortened version of the EORTC QLQ-C30 [81], one of the most widely used QoL questionnaires in oncology. Because the QLQ-C30 contains 30 items, was not initially developed for use in PC, and some of its content has been perceived as inappropriate by patients receiving PC, the QLQ-C15-PAL was developed, is well-validated and cross-culturally adapted, and is considered a core PC questionnaire. The QLQ-C15-PAL includes those elements of the QLQ-C30 identified as most relevant and important for PC, i.e. physical and emotional function, pain, fatigue, nausea/vomiting, appetite, dyspnea, constipation, sleeping difficulties, and overall QoL. All but the QoL item are scored on categorical scales from 1 (not at all) to 4 (very much) and transformed to a scale of 0–100. The Norwegian version has been validated and used in several PC studies [63, 82, 83].

### QLQ-INFO25

The QLQ-INFO25 (European Organisation of Research and Treatment of Cancer Quality Questionnaire-Information) [84] was developed to evaluate cancer patients’ perception of information received during different phases of care and has been cross-culturally validated according to the EORTC guidelines for module development [85]. The module has four multi-item scales—information about the disease (four items), medical tests (three items), treatment (six items), and other services (four items)—and eight single items, scored on categorical scales from 1 (not at all) to 4 (very much) and transformed to scales of 0–100. For the purposes of PALLiON, we have decided to delete one single item regarding sexuality as this may be perceived as inappropriate by patients in the last stages of life. The psychometric properties of the QLQ-INFO25 were confirmed, demonstrating a reliable and valid self-reported instrument. All items can be combined to generate a single score (α > 0.90). The module discriminated among groups based on gender, age, education, levels of anxiety and depression, information wishes and satisfaction, and was well suited for cross-cultural observational and intervention studies and together with, for example, the QLQ-C15-PAL scale.

### PHQ-9

Depressive symptoms will be assessed using the PHQ-9 [72], a self-report questionnaire commonly used in medically ill samples [4, 86, 87], and has proven good validity and sensitivity. The nine PHQ-items correspond to the DSM-5 diagnostic criteria for major depressive disorder and assess the frequency at which the symptoms have been bothersome during the past two weeks: 0 = “not at all”; 1 = “several days”; 2 = “more than half the days”; and 3 = “nearly every day”. To be classified as depressed according to the DSM-5 criteria, five out of nine criteria, including anhedonia and/or depressed mood, have to be present and scored as bothersome for at least “more than half the days” or “on some days”.

### GAD-7

The Generalized Anxiety Disorder 7-item (GAD-7) questionnaire is based on the DSM criteria for a generalized anxiety disorder [88]. It consists of seven items on general anxiety commonly experienced by cancer patients, such as tension and worrying in the past 2 weeks, scored on a 4-point Likert scale (0 = not at all, 3 = almost every day). The total score is in the range of 0–21. GAD-7 has been shown to have good reliability, as well as good criterion, construct, factorial and procedural validity, with a suggested cut point that optimized sensitivity (89%) and specificity (82%). GAD-7 is not defined as an outcome in PALLiON, but the items in GAD-7 address frequently experienced psychological symptoms in patients with cancer, which is the rationale for choosing this instrument.

### Caregiver-reported outcome measures background variables

This baseline caregiver form has eight variables concerning sociodemographic variables, i.e. date of birth, date of consent, marital, living, level of education and working status, relation to patient, and current chronic diseases. These variables are not included in subsequent caregiver forms.

### FAMCARE

Although patient satisfaction with care has been shown to be amenable to change with PC interventions, only a few intervention studies have demonstrated a positive effect of specific PC interventions on caregivers’ health and QoL [17, 21]. The Family Satisfaction with End-of-Life Care (FAMCARE) scale [89] is a 20-item self-report measure that was developed to measure family satisfaction with PC for cancer. The questionnaire asks for the degree to which family members are content with the healthcare provider behaviors directed toward the patient and themselves and can be administered during the disease trajectory or at some point after a patient’s death. A Norwegian C-RCT showed that caregivers to patients with cancer receiving specialized PC reported higher satisfaction with EoL care on FAMCARE than caregivers in the control group [21].

### SF-36

SF-36 is a brief, yet comprehensive generic measure of subjective health and QoL [90]. The 36 items can be summed into eight multi-item scales measuring physical functioning, bodily pain, general health perception, mental health, role limitations due to physical problems, role limitations due to emotional problems, social function, and vitality. An additional item reports health transition over the past year. Items are scored and transformed to scales of 0–100 for ease of interpretation. Extensive reference data exist from Norwegian population surveys [91, 92], facilitating comparisons across populations.

In line with the definition of PC, caregiver follow-up is essential. As we believe in the study’s beneficial effects on caregiver perception of care, we will follow the caregiver twice after the patient is dead. The questionnaire packet will be sent by mail at 6 and 12 months after the patient’s death, with a specific consent form inserted.

### Organization

Oslo University Hospital is the Sponsor, i.e. the main responsible party for this study. Initial study planning was conducted in collaboration with St Olav’s Hospital, Trondheim, particularly related to setting up the Trial Office and preparing the adaptation, installation, and functional aspects of Eir and in the development of the PCCPs.

The local principal investigators and study nurses at the participating hospitals are responsible for patient inclusion and registration of clinical data in the e-CRF every 2 months. The Trial Office is located at St Olav’s Hospital, Trondheim, with study personnel being responsible for the overall data management and study administration. This includes administration of the e-CRFs, sending out and scanning the paper questionnaires to patients and caregivers, regular monitoring of data quality, and preparation of the final data files.

Patients included in the study are covered by the Patient Injuries Act. No particular insurance applies to relatives. Study participation is not likely to cause any harm to participants, be it patients or caregivers. Study results will be published in peer-reviewed journals. Authorship is based upon the Vancouver rules. All manuscripts will be prepared by the researchers.

## Trial status

Inclusion of patients and caregivers is ongoing. Recruitment started in March 2017. It took longer than anticipated to finish the comprehensive educational program at the intervention sites. As completion of this program was requested before the start of patient inclusion, it became evident that a prolongation of the study timeline was necessary. This has been updated in ClinTrials.gov and was approved by the Ethical Committee South East Norway as a study amendment in November 2019. The final date for study recruitment is 31 December 2020, or before if 680 patients have been included, to secure that the power requirements are met. The final date for patient follow-up is 31 December 2021, or before if the last included patient has been followed for 1 year or until death. Caregivers will be assessed at 6 and 12 months after the patient is dead, if they consent to continue in the study. Thus, caregiver follow-up will continue in 2022.

## Supplementary information

**Additional file 1.** SPIRIT checklist.

## Data Availability

The datasets generated and/or analyzed during the current study are available from the corresponding author on reasonable request.

## Abbreviations

BPI: Brief Pain Inventory
CONSORT: Consolidated Standards of Reporting Trials
C-RCT: Cluster-randomized controlled trial
CTCAE: Common Terminology Criteria for Adverse Events
CV: Coefficient of variation
EAPC: European Association for Palliative Care
e-CRF: Electronic case report form
EoL: End-of-life
EORTC QLQ-INFO25: European Organisation of Research and Treatment of Cancer Quality Questionnaire-Information
EORTC: European Organisation for Research and Treatment of Cancer
EPA: European Pathway Association
e-PROM: Electronic patient reported outcome measure
ESAS: Edmonton Symptom Assessment System
F-36: Short Form-36
FAMCARE: Family Satisfaction with End-of-Life Care
FDA: Food and Drug Administration
GAD: General anxiety disorder
GT: Gamma-glutamyltransferase
HCP: Healthcare professionals
ICC: Intra-cluster correlation coefficient
KPS: Karnofsky Performance Status
LD: Lactate-dehydrogenase
NRS: Numerical rating scale
NSCLC: Non–small cell lung cancer
NTNU: Norwegian University of Science and Technology
PC: Palliative care
PCCP: Patient-centered care pathway
PHQ-9: Patient Health Questionnaire-9
PRO: Patient-reported outcome
PROM: Patient-reported outcome measure
QLQ-C15-PAL: European Organisation of Research and Treatment of Cancer Quality Of Life Questionnaire Core 15 Palliative
QoL: Quality of life
RCT: Randomized controlled trial
REC: Regional Committee for Medical and Health Research Ethics
RECIST: Response Evaluation Criteria In Solid Tumors
SCO: American Society of Oncology
SCP: Standardized care pathway
SGA: Subjective Global Assessment for nutritional status
SPIRIT: Standard Protocol Items
WHO: World Health Organization
